# Peri‐Implant Soft Tissue Response to Monolithic Zirconia Versus Porcelain‐Fused‐to‐Metal Implant Crowns: Histological and Clinical Outcomes of an RCT

**DOI:** 10.1111/jcpe.70169

**Published:** 2026-07-15

**Authors:** Yifan Zhang, Sven Mühlemann, Aspasia Pachiou, Stephanie Astrid Stalder, Ronald E. Jung, Ye Lin, Daniel S. Thoma

**Affiliations:** ^1^ Department of Implantology Peking University School and Hospital of Stomatology, National Center of Stomatology, National Clinical Research Center for Oral Diseases, National Engineering Research Center of Oral Biomaterials and Digital Medical Devices Beijing China; ^2^ Clinic of Reconstructive Dentistry, Center of Dental Medicine, University of Zurich Zurich Switzerland; ^3^ Private Practice Zurich Switzerland; ^4^ Department of Periodontology Research Institute for Periodontal Regeneration, Yonsei University College of Dentistry Seoul Republic of Korea

**Keywords:** dental implants, histology, monolithic zirconia, peri‐implant soft tissue, porcelain fused to metal

## Abstract

**Objective:**

To assess the peri‐implant soft‐tissue and hard‐tissue response to monolithic zirconia (ZrO_2_) compared to porcelain‐fused‐to‐metal implant crowns (PFMs).

**Methods:**

This report constitutes a pre‐specified secondary analysis of this RCT. Eighty‐three patients rehabilitated with single‐tooth implants in the molar region were originally randomly assigned to receive either a ZrO_2_ or a PFM restoration. At 6 months, clinical parameters (plaque control record, probing depth, bleeding on probing, width of keratinised mucosa and marginal bone level changes) were recorded at the implant site and the adjacent natural tooth. In addition, a peri‐implant soft‐tissue biopsy specimen was harvested between the implant site and the adjacent tooth. The number of inflammatory cells and fibroblasts/fibrocytes was evaluated within four regions of interest (oral epithelium, sulcular epithelium, junctional epithelium, connective tissue). Descriptive statistics were arrived at for all outcomes, and differences between prosthetic materials, sites and timepoints were analysed using linear mixed‐effects models.

**Results:**

The present exploratory secondary analysis included 67 patients (37 ZrO_2_; 30 PFM) with complete 6‐month clinical, radiographic and histological data. Clinical parameters at both implant and adjacent tooth sites remained generally stable from baseline to 6 months, with limited marginal bone level changes and comparable outcomes between groups. Histologically, mixed‐effects model analysis showed that the tissue region significantly affected both the inflammatory infiltrate and the fibroblast density (*p* < 0.001). The crown materials did not substantially influence the histological outcomes. The distribution of inflammatory infiltrate differed between tooth and implant sites, with the highest values in the sulcular epithelium around implants and in the junctional epithelium around teeth. Fibroblast density was highest in the junctional epithelium at both sites.

**Conclusion:**

Monolithic zirconia and PFM implant‐supported single crowns resulted in comparable clinical and radiographic outcomes. The peri‐implant soft‐tissue response to the two restorative materials showed a greater effect of the tissue region, while the material itself had only negligible effect.

**Trial Registration:**

ClinicalTrials.gov identifier: NCT02272491

## 1 Introduction

The integrity of the peri‐implant tissue is considered a key determinant of long‐term implant stability (Stefanini et al. 2023). Although the peri‐implant mucosa resembles that of natural teeth in its general architecture, histological differences—such as a reduced vascularity and the parallel orientation of collagen fibres—have been described around implants (Ivanovski and Lee 2018). Experimental and human histopathological studies indicate that inflammatory lesions around implants may extend more apically and involve larger inflammatory infiltrates than periodontal lesions, potentially contributing to the more rapid progression pattern observed in peri‐implantitis (Carcuac and Berglundh 2014; Salvi et al. 2023).

Various factors may influence the integrity, the biological response and the long‐term stability of the peri‐implant mucosa. These include systemic conditions such as smoking, radiation therapy (Karbach et al. 2009), diabetes and other systemic diseases (Gómez‐Moreno et al. 2015), as well as local factors including inadequate oral hygiene (Ferreira et al. 2006), prosthetic designs that impair plaque control (Heitz‐Mayfield et al. 2011) and excess luting cement (Linkevicius et al. 2013). Biofilm accumulation remains the primary aetiological factor for peri‐implant mucositis, which is an inflammatory lesion of the peri‐implant mucosa without progressive bone loss and considered a precursor of peri‐implantitis (Heitz‐Mayfield and Salvi 2018). In addition, the material characteristics of the implant and restorative components in contact with the peri‐implant mucosa may also influence the biological response of peri‐implant soft tissues.

Monolithic zirconia restorations, in general cemented extraorally on titanium bases, have become a widely used restorative option for implant‐supported single crowns in the posterior region. Various biological, clinical and technical characteristics have contributed to their widespread use for implant‐supported single crowns. This predominantly includes reduced risk of chipping, compatibility with digital workflows (Derksen et al. 2023) and low plaque accumulation, thereby contributing to reduced bacterial colonisation (Małysa et al. 2025; Tang et al. 2023). Although (pre)clinical data show that PFM materials, most likely due to the veneering, lead to greater inflammatory reaction than zirconia (Abrahamsson et al. 1998), clinical data complemented by histological analyses are scarce.

The aim of the present study was to assess the peri‐implant soft‐and hard‐tissue response to monolithic zirconia (ZrO_2_) and porcelain‐fused‐to‐metal (PFM) implant crowns at 6 months. As a pre‐specified secondary analysis of a parent RCT (Mühlemann et al. 2020; Zumstein et al. 2023), this report presents the histological findings together with clinical and radiographic outcomes, including paired biopsies from peri‐implant and adjacent periodontal tissues.

## 2 Materials and Methods

### 2.1 Study Design and Patient Selection

The study was designed as a prospective randomised controlled clinical trial with two parallel study groups. The clinical protocol was approved by the local ethics committee (PB_2016‐01977) and registered at ClinicalTrials.gov (NCT02272491). The research protocol followed the Helsinki Declaration of 1975 (revised in 2000 and 2008), and the patients provided informed consent to participate in the study. The study was reported in accordance with the CONSORT 2025 statement (Hopewell et al. 2025).

Eighty‐three partially edentulous patients in need of a single implant‐supported crown in at least one maxillary or mandibular molar site were recruited at the Clinic of Reconstructive Dentistry, University of Zurich, Switzerland. Detailed eligibility criteria have been published previously (Mühlemann et al. 2020). Briefly, partially edentulous adults requiring a single implant‐supported molar crown were included.

### 2.2 Clinical and Laboratory Procedures

All sites received titanium–zirconium narrow‐diameter implants (Straumann Standard Plus SLActive RN, Roxolid, 3.3 mm diameter, Institut Straumann AG). The implants were placed according to the manufacturer's recommendation. All implants were left for transmucosal healing.

An implant impression was taken 3–6 months after implant placement. At this timepoint, patients were randomly allocated to one of the treatment modalities according to a computer‐generated randomisation list. Allocation concealment was ensured by an independent person not involved in the study using an electronic data capture system (secuTrial, Clinical Trials Center, University Hospital Zurich). Patients received one of the following randomly assigned implant crowns:

#### 2.2.1 ZrO_2_ Group

Monolithic 3 mol% yttria partially stabilised zirconia crown (Lava Plus, 3 M, Seefeld) bonded to a titanium base abutment (Straumann RN Variobase, 1 mm mucosal height).

#### 2.2.2 PFM Group

Porcelain‐fused‐to‐metal (PFM) crown consisting of a gold abutment (Straumann RN synOcta cast gold abutment) with a cast high noble gold alloy (V‐Classic, Cendres Métaux) and feldspathic veneering ceramic (Creation CC, Willi Geller, Klema).

All crowns were fabricated by one experienced master dental technician. In the test group, the submucosal part of the crown was left unstained to maintain a highly polished zirconia surface in contact with the peri‐implant mucosa. The titanium base abutment was air‐abraded with 50‐μm aluminium oxide particles from a distance of 1 cm for 15 s at 2.8 bar pressure. The abutment and crown were subsequently cleaned with ethanol and treated with a primer. The crown was then luted to the abutment using a chemically curing composite cement (Multilink Hybrid Abutment, Ivoclar Vivadent). In the control group, conventional fabrication included the lost‐wax technique for casting the high noble gold core, followed by manual layering of the veneering ceramic and ceramic firing (Austromat M, DEKEMA) according to the manufacturers' instructions.

All implant crowns were screw‐retained with a torque of 35 N cm. The screw access hole was filled with Teflon tape and sealed with a composite filling (Filtek, 3M).

### 2.3 Clinical Examination and Outcome Measures

#### 2.3.1 Clinical Examination

All patients were recalled for the baseline examination 1–2 weeks after crown insertion (BL) and again at the 6‐month follow‐up (6 m‐FU). Two calibrated examiners performed all clinical measurements. Inter‐examiner reliability was assessed in 10 pilot patients: Cohen's kappa for BOP = 0.82; ICC for probing depth = 0.93 (95% CI 0.85–0.97).

The following clinical parameters were recorded around each implant and the mesial adjacent tooth at six sites using a periodontal probe (PCB 12, Hu‐Friedy, Germany):

- plaque control record (PCR)
- probing depth (PD)
- bleeding on probing (BOP)
- width of keratinised mucosa (KM; measured only at the buccal aspect)

Peri‐implant tissue conditions at the 6‐month follow‐up were categorised as peri‐implant health or peri‐implant mucositis. Peri‐implant mucositis was defined as the presence of bleeding on probing (BOP > 0) in the absence of progressive marginal peri‐implant bone loss, in accordance with the definition proposed by Heitz‐Mayfield and Salvi (2018).

#### 2.3.2 Radiographic Examination

Standardised digital periapical radiographs (Digora Optime, Soredex) were taken at BL and at 6 m‐FU using the paralleling technique. Radiographs were analysed using an open‐source image analysis software (ImageJ, National Institutes of Health). A calibrated independent examiner measured the distance between the implant shoulder and the first bone‐to‐implant contact at the mesial and distal aspects to the nearest 0.1 mm. Intra‐examiner reliability was evaluated on 15 randomly selected radiographs measured twice at an interval of ≥ 4 weeks: ICC = 0.96 (95% CI 0.90–0.98).

Implant length and the distance between implant threads were used for calibration. Changes in marginal bone level (MBL) were calculated from 6 m‐FU to BL. Bone loss was defined as a positive change in MBL.

### 2.4 Biopsy Harvesting and Histological Analysis

**FIGURE 1:**
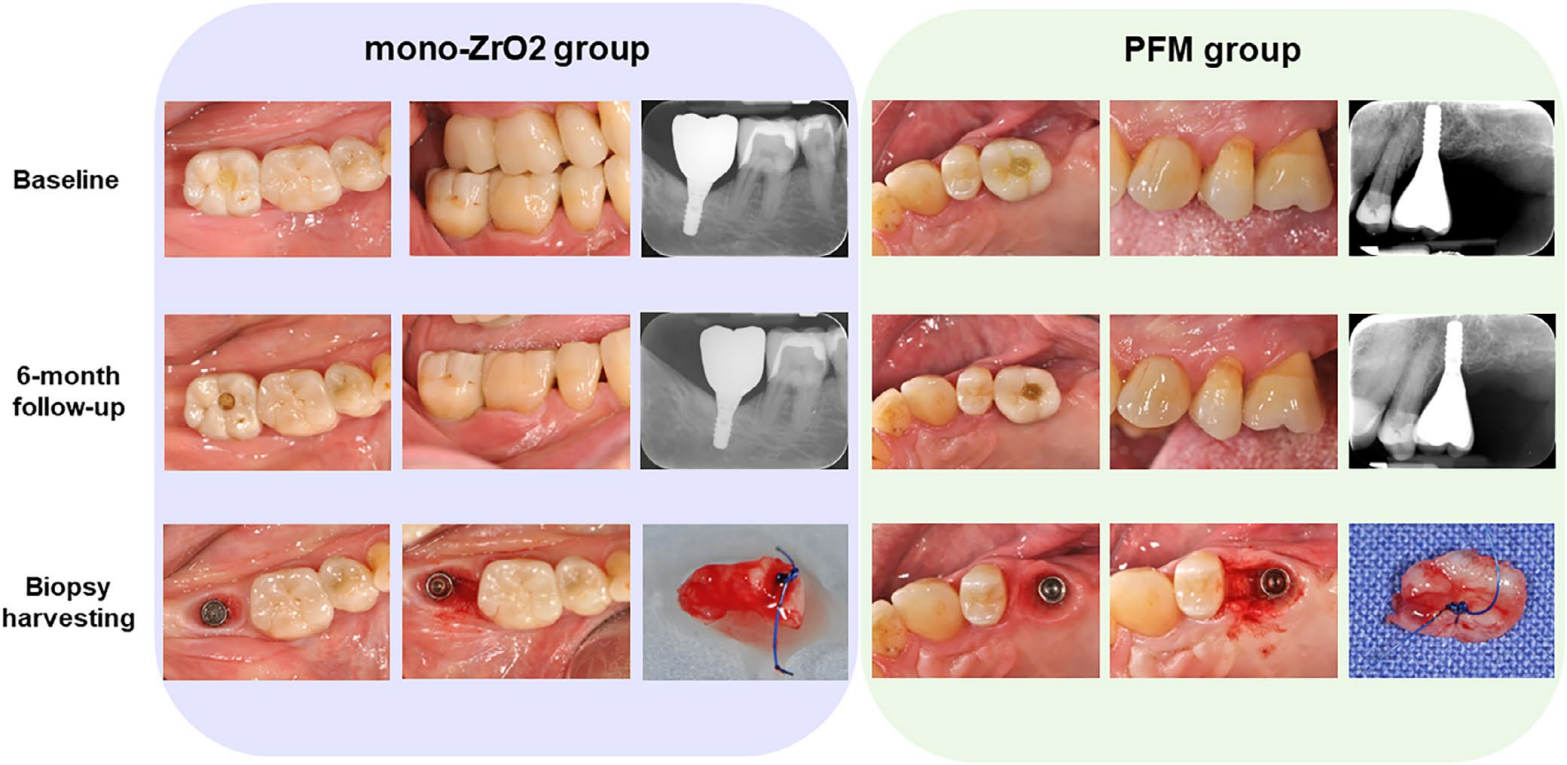
Representative clinical, radiographic and biopsy‐harvesting images from the mono‐ZrO_2_ and PFM groups at baseline and 6‐month follow‐up.

At the 6‐month follow‐up, a biopsy of the peri‐implant mucosa and the adjacent gingiva of the natural tooth inter‐proximally provided that at least 2 mm of keratinised tissue was present. The biopsy had a rough dimension of 2 mm in bucco‐oral width and extended from the sulcus of the natural tooth to the implant crown. The vertical dimension extended from the mucosal/gingival margin to the bone crest (Figure 1).

**FIGURE 2:**
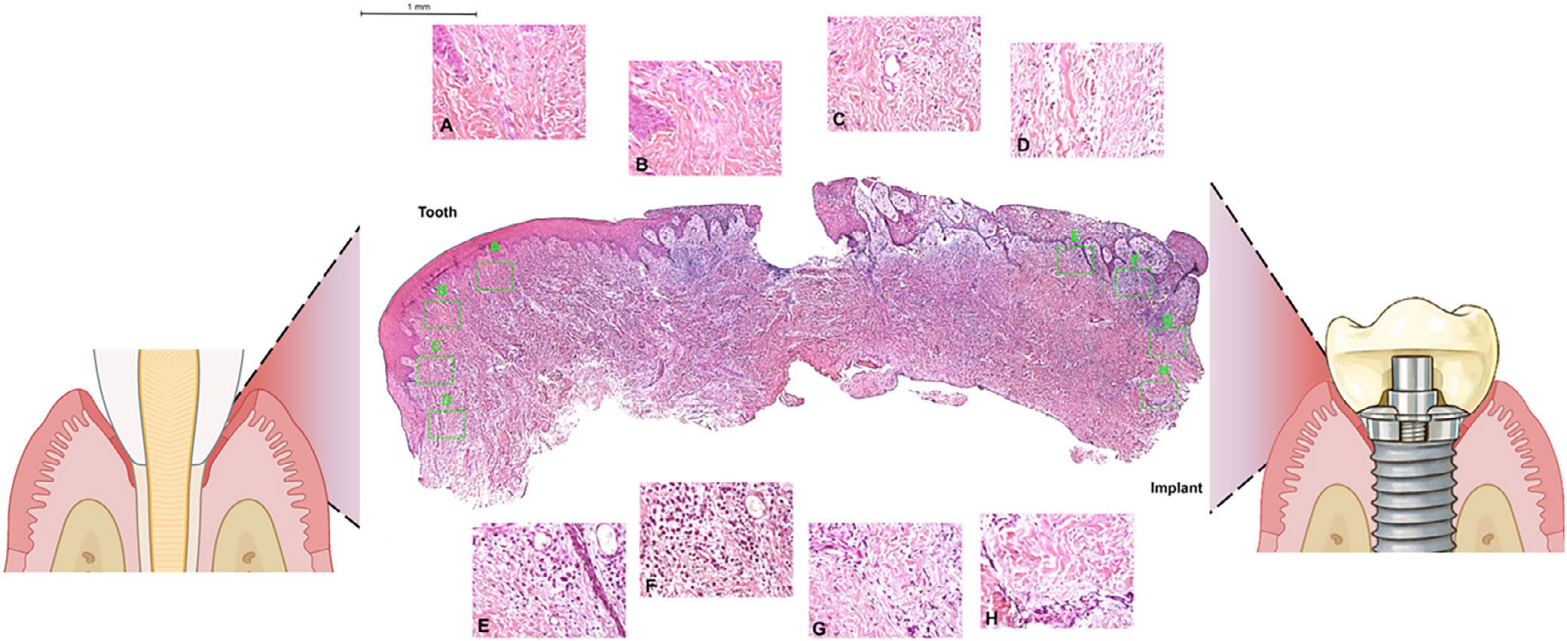
Schematic diagram of biopsy harvesting and histological analysis. Four regions of interest were analysed: (A and E) oral epithelium; (B and F) sulcular epithelium; (C and G) junctional epithelium; (D and H) supracrestal connective tissues.

Biopsy specimens were fixed for 48 h in 4% buffered formalin, dehydrated, infiltrated with xylol and paraffin and sectioned using a paraffin microtome (MICROM, Medite GmbH). Specimens were oriented with the coronal margin upward and embedded in a standardised bucco‐oral plane. Three non‐serial sections were obtained from each block at depths of approximately 50, 150 and 300 μm from the outer surface. The section showing the most complete representation of all four regions of interest was selected for analysis. Representative histological sections illustrating the four pre‐defined regions of interest are shown in Figure 2. Sections were stained with haematoxylin–eosin.

A blinded examiner performed a semi‐quantitative analysis of fibroblasts and inflammatory cells in each region. Intra‐examiner reliability (10 specimens re‐evaluated after ≥ 4 weeks): ICC = 0.89 (95% CI 0.74–0.96) for inflammatory infiltrate; ICC = 0.91 (95% CI 0.78–0.97) for fibroblast density. The percentage area of each subgroup was calculated using the image analysis software (LAS V4.3, Leica).

Four regions of interest were analysed (Figure 2):

- oral epithelium
- sulcular epithelium
- junctional epithelium
- supracrestal connective tissue

### 2.5 Statistical Analysis

The primary objective of the parent RCT was to evaluate technical complications over a 5‐year follow‐up period; therefore, sample size calculation was based on this endpoint (Mühlemann et al. 2020). The present study represents a pre‐specified secondary analysis of 6‐month clinical and histological outcomes. Histological assessment of peri‐implant soft tissues was defined as a secondary biological outcome in the original protocol, and findings should be interpreted as exploratory.

Statistical analyses were performed in R (version 4.6.0; R Foundation for Statistical Computing, Vienna, Austria) using RStudio (version 2026.04.0+526) and the packages dplyr, tidyverse, lmerTest, ggplot2, emmeans and tidyr. Descriptive data were reported as mean ± SD, median (Q1–Q3) or frequencies and percentages, as appropriate.

Histological outcomes were analysed using linear mixed‐effects models with subject as a random effect to account for repeated measurements. Fixed effects included restorative material, site, histological region and their interactions. For BOP, mean values from mesial, central and distal measurements were analysed as continuous variables using a mixed‐effects model including site, material, time and their interactions. Post hoc pairwise comparisons were performed when appropriate, with adjustment for multiple testing. Sensitivity analyses additionally adjusted for age, baseline keratinised tissue width and baseline peri‐implant mucositis status.

Within‐patient tooth–implant comparisons were assessed using Wilcoxon signed‐rank and rank‐sum tests. Statistical significance was set at *p* < 0.05.

## 3 Results

**FIGURE 3:**
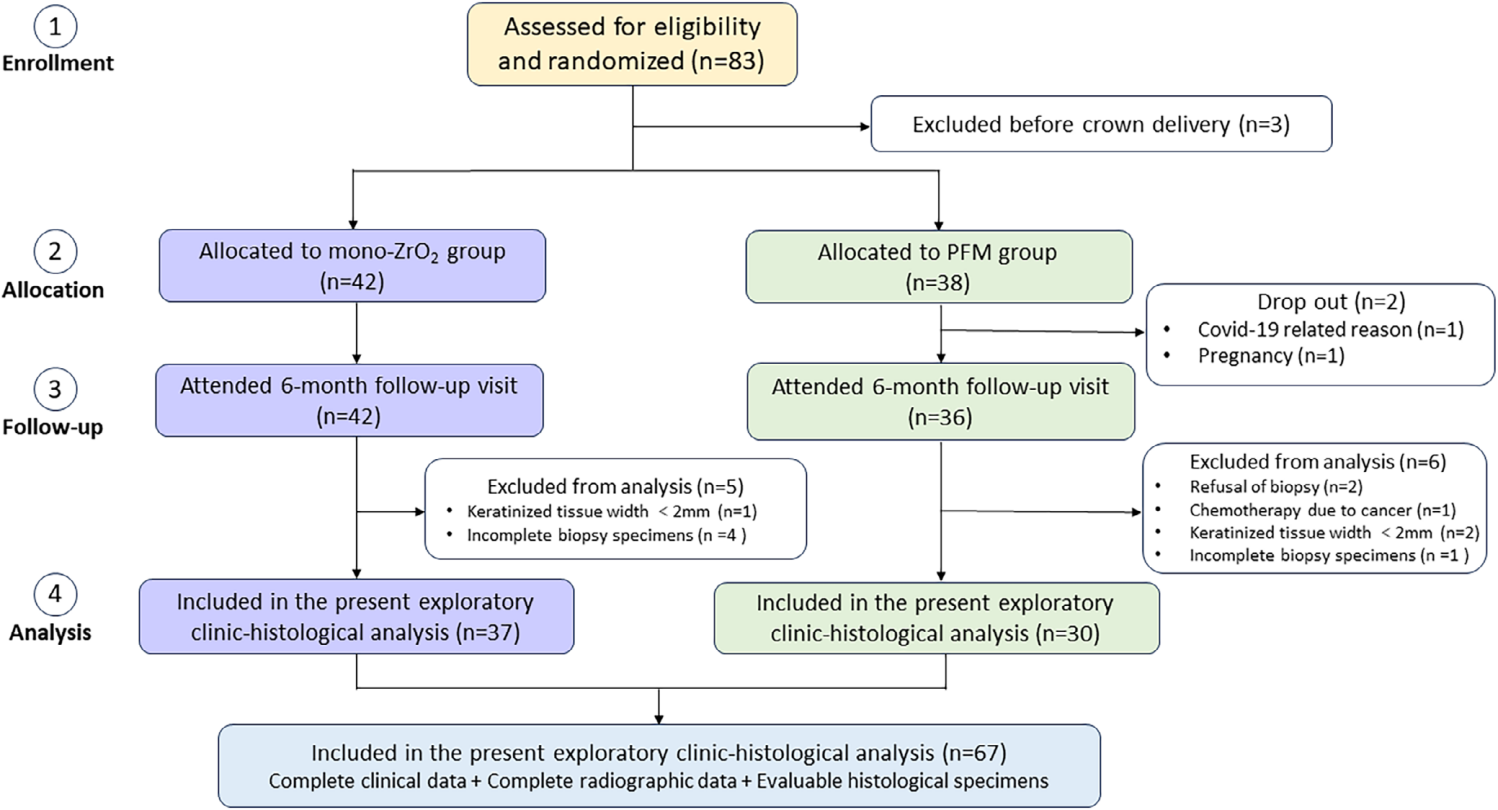
CONSORT flow diagram illustrating patient enrolment, follow‐up, exclusions and inclusion in the present exploratory clinico‐histological analysis.

Of the 83 patients originally randomised in the parent RCT, 67 were included in the present exploratory secondary analysis. Patient flow and reasons for exclusion are presented in Figure 3. This sub‐population (67 patients) consisted of 37 males and 30 females with a mean age of 65.3 years (range 40.5–91.6 years). Thirty‐seven patients received monolithic zirconia (ZrO_2_) crowns and 30 received PFM crowns.

Clinical parameters at both adjacent tooth and implant sites remained generally stable from baseline to 6 months (Table 1). At baseline, peri‐implant health and peri‐implant mucositis were present in 55.2% (37/67) and 44.8% (30/67) of implant sites, respectively; corresponding values at 6 months were 52.2% (35/67) and 47.8% (32/67). Keratinised tissue width increased slightly at the adjacent tooth in both the PFM (0.24 ± 0.83 mm) and ZrO_2_ groups (0.34 ± 1.04 mm), while the values at the implant sites remained largely unchanged (PFM: 0.06 ± 0.79 mm; ZrO_2_: 0.05 ± 0.89 mm). Probing depth showed minor variations, with a slight increase at the implant site in the PFM group (0.34 ± 0.50 mm) and stable values in the ZrO_2_ group (−0.01 ± 0.67 mm). Plaque control record (PFM: −0.01 ± 0.10; ZrO_2_: 0.02 ± 0.19) and bleeding on probing (PFM: 0.06 ± 0.28; ZrO_2_: 0.01 ± 0.25) remained low with minimal changes over time.

**TABLE 1:** Results of the clinical parameters at baseline and at 6‐month follow‐up.^a^

	Baseline		6 months		Difference: 6‐month follow up‐baseline	
	Mean ± SD	Median (Q1–Q3)	Mean ± SD	Median (Q1–Q3)	Mean ± SD	Median (Q1–Q3)
PD (mm)						
PFM (tooth)	2.39 ± 0.50	2.33 (2.17–2.67)	2.51 ± 0.52	2.50 (2.08–2.83)	0.12 ± 0.42	0.17 (−0.33 to 0.33)
ZrO_2_ (tooth)	2.35 ± 0.41	2.33 (2.00–2.67)	2.38 ± 0.54	2.33 (2.00–2.67)	0.03 ± 0.41	0.00 (−0.33 to 0.33)
PFM (implant)	3.01 ± 0.62	2.83 (2.67–3.46)	3.35 ± 0.59	3.25 (2.83–3.62)	0.34 ± 0.50	0.33 (0.00–0.67)
ZrO_2_ (implant)	3.03 ± 0.55	3.00 (2.67–3.33)	3.02 ± 0.56	2.83 (2.67–3.33)	−0.01 ± 0.67	−0.17 (−0.50 to 0.50)
PCR						
PFM (tooth)	0.09 ± 0.14	0.00 (0.00–0.17)	0.16 ± 0.22	0.08 (0.00–0.17)	0.07 ± 0.27	0.00 (0.00–0.17)
ZrO_2_ (tooth)	0.09 ± 0.16	0.00 (0.00–0.17)	0.11 ± 0.16	0.00 (0.00–0.17)	0.02 ± 0.20	0.00 (0.00–0.17)
PFM (implant)	0.03 ± 0.09	0.00 (0.00–0.00)	0.02 ± 0.06	0.00 (0.00–0.00)	−0.01 ± 0.10	0.00 (0.00–0.00)
ZrO_2_ (implant)	0.04 ± 0.09	0.00 (0.00–0.00)	0.06 ± 0.16	0.00 (0.00–0.00)	0.02 ± 0.19	0.00 (0.00–0.00)
BOP						
PFM (tooth)	0.08 ± 0.11	0.00 (0.00–0.17)	0.14 ± 0.15	0.17 (0.00–0.29)	0.07 ± 0.16	0.00 (0.00–0.17)
ZrO_2_ (tooth)	0.07 ± 0.11	0.00 (0.00–0.17)	0.07 ± 0.12	0.00 (0.00–0.17)	0.00 ± 0.16	0.00 (0.00–0.00)
PFM (implant)	0.09 ± 0.14	0.00 (0.00–0.17)	0.14 ± 0.21	0.08 (0.00–0.17)	0.06 ± 0.28	0.00 (−0.13 to 0.17)
ZrO_2_ (implant)	0.14 ± 0.18	0.17 (0.00–0.17)	0.16 ± 0.20	0.00 (0.00–0.33)	0.01 ± 0.25	0.00 (−0.17 to 0.17)
KM (mm)						
PFM (tooth)	2.88 ± 1.30	3.00 (2.00–4.00)	3.12 ± 1.20	3.00 (3.00–4.00)	0.24 ± 0.83	0.00 (0.00–0.00)
ZrO_2_ (tooth)	3.42 ± 1.37	3.00 (3.00–4.00)	3.77 ± 1.02	4.00 (3.00–4.00)	0.34 ± 1.04	0.00 (0.00–1.00)
PFM (implant)	2.52 ± 1.30	2.00 (2.00–3.00)	2.58 ± 1.12	2.00 (2.00–3.00)	0.06 ± 0.79	0.00 (0.00–0.00)
ZrO_2_ (implant)	2.50 ± 1.05	2.00 (2.00–3.00)	2.55 ± 1.03	3.00 (2.00–3.00)	0.05 ± 0.89	0.00 (−1.00 to 1.00)
MBL (mm)						
PFM	2.00 ± 0.53	1.91 (1.47–2.43)	2.12 ± 0.83	2.15 (1.31–2.78)	0.13 ± 0.53	0.10 (−0.16 to 0.63)
ZrO_2_	2.09 ± 0.37	1.93 (1.76–2.44)	2.31 ± 0.96	2.21 (1.69–2.63)	0.22 ± 0.79	0.14 (−0.26 to 0.52)

Abbreviations: BOP, bleeding on probing; KM, width of keratinised mucosa; MBL, marginal bone level; PCR, plaque control record; PD, probing pocket depth; PFM, porcelain fused to metal; SD, standard deviation; ZrO_2_, zirconia crown.

^a^ Data are presented for the subgroup of patients with complete 6‐month clinical, radiographic and evaluable histological data included in the present exploratory secondary analysis (*n* = 67).

Radiographic marginal bone level changes were limited (PFM: 0.13 ± 0.53 mm; ZrO2: 0.22 ± 0.79 mm). Overall, clinical outcomes were comparable between the two restorative groups.

Mixed‐effects model analysis of BOP (Table 2) showed a significant effect of site, with higher BOP values at implant sites than at adjacent teeth (*p* = 0.026). No significant effects were observed for the restorative material (*p* = 0.860) or time (*p* = 0.070).

**TABLE 2:** Mixed‐effects model evaluating factors associated with BOP and histological outcomes.^a^

Outcome	Factor/contrast	*F*	Estimate	95% CI	*p*
BOP	Site: Implant versus tooth	5.06	0.043	0.005 to 0.081	0.026*
	Material: Mono ZrO_2_ versus PFM	0.03	−0.004	−0.043 to 0.036	0.860
	Time: 6 months versus baseline	3.31	0.035	−0.003 to 0.073	0.070
	Material × Site	3.85	—	—	0.051
	Site × Time	0.00	—	—	0.978
	Material × Time	1.83	—	—	0.178
	Site × Material × Time	0.07	—	—	0.794
Inflammatory infiltrate (%)	Material: PFM versus ZrO_2_	0.24	0.233	−0.718 to 1.180	0.627
	Site: Implant versus tooth	3.70	0.580	−0.013 to 1.170	0.055
	Region	11.95	—	—	< 0.001***
	Material × Site	0.11	—	—	0.739
	Material × Region	0.36	—	—	0.779
	Site × Region	11.53	See Table S1	See Table S1	< 0.001***
	Material × Site × Region	0.48	—	—	0.695
Fibroblast density (%)	Material	1.49	—	—	0.226
	Site	0.17	—	—	0.678
	Region	15.23	—	—	< 0.001***
	Material × Site	8.23	See Table S1	See Table S1	0.004**
	Material × Region	0.37	—	—	0.778
	Site × Region	4.50	See Table S1	See Table S1	0.004**
	Material × Site × Region	1.10	—	—	0.347

Abbreviations: BL, baseline; BOP, bleeding on probing; CT, supracrestal connective tissue; JE, junctional epithelium; OE, oral epithelium; PFM, porcelain‐fused‐to‐metal; SE, sulcular epithelium; ZrO_2_, zirconia crown.

^a^ Estimates and 95% CIs are reported for principal two‐level contrasts. For multi‐level factors and significant interaction terms, detailed estimated marginal means and post hoc contrasts are provided in Table S1.

*p < 0.05; **p < 0.01; ***p < 0.001.

**FIGURE 4:**
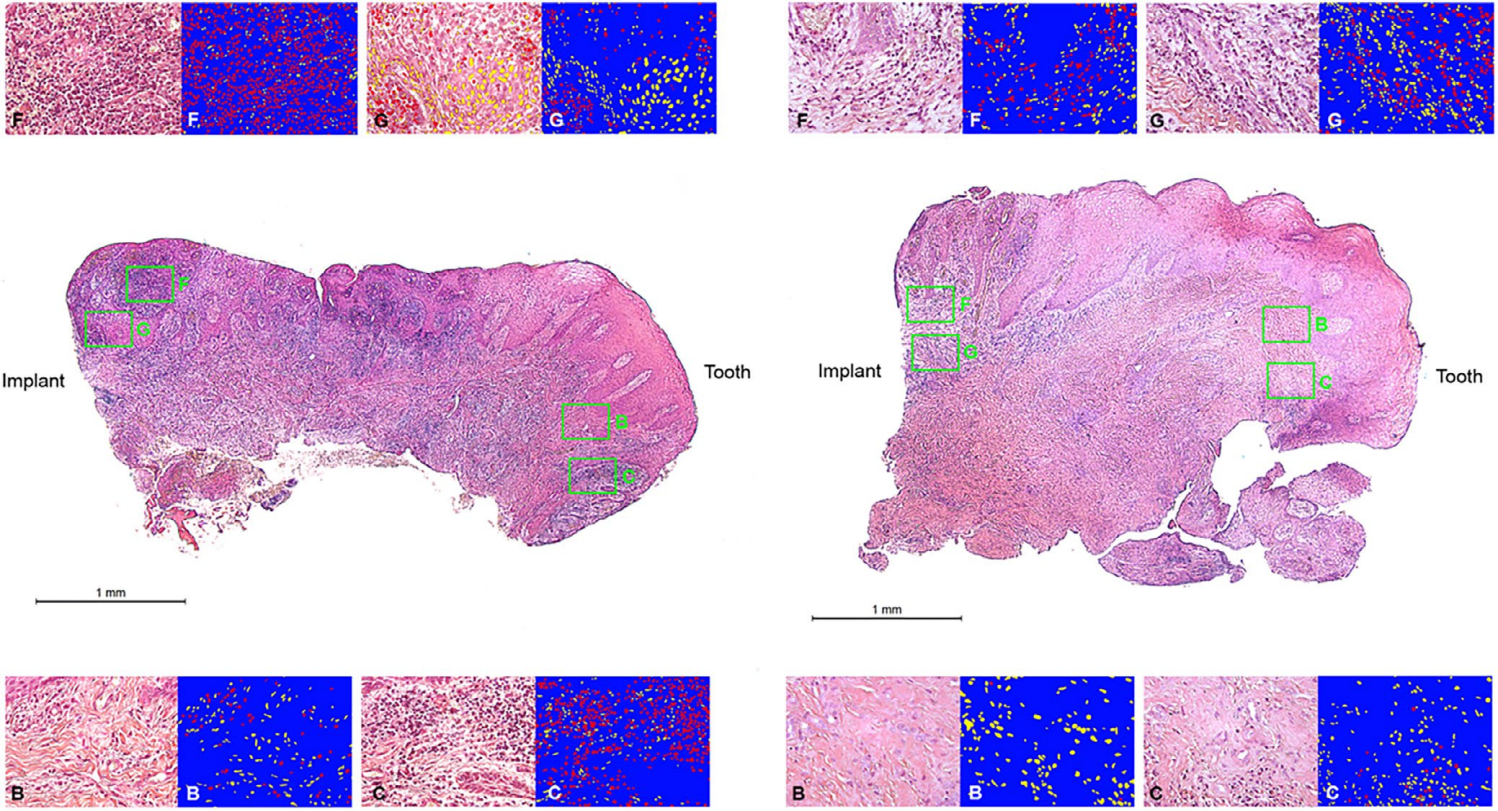
Histological analysis comparing PFM (left) and ZrO_2_ (right), showing the inflammatory cells of compartments B/F and C/G. Cells are marked in yellow (fibroblasts) and in red (inflammatory cells).

**FIGURE 5:**
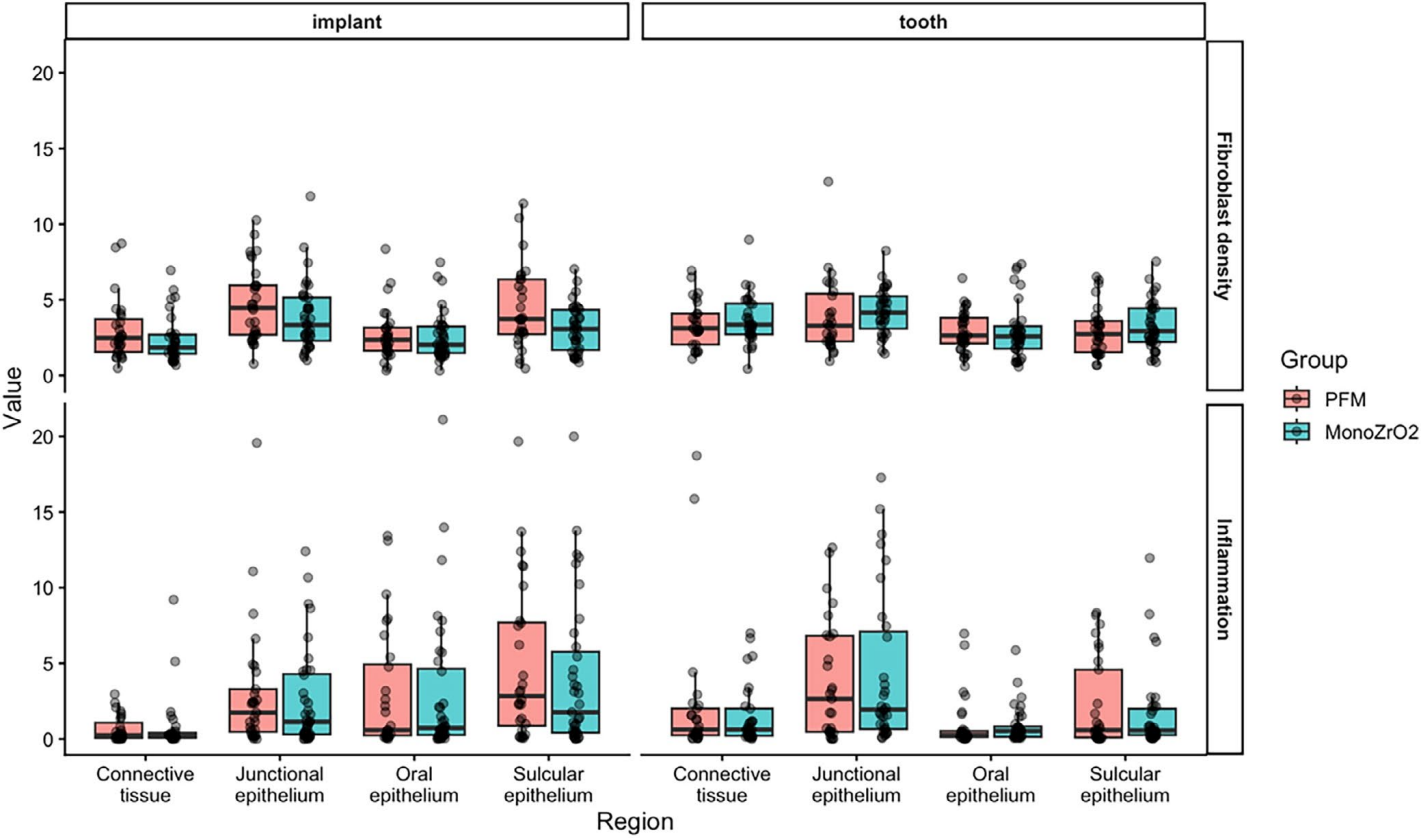
Distribution of histological inflammation and fibroblast density across regions at implant and tooth sites for PFM and zirconia (ZrO_2_) restorations. Boxplots represent median and interquartile range, with individual observations overlaid. Regional differences were more pronounced than material‐related differences for both outcomes.

Histologically, both inflammatory infiltrate and fibroblast density varied significantly across tissue regions (Table 2, Figures 4 and 5). Tissue region significantly influenced inflammatory infiltrate (*p* < 0.001), with different distribution patterns between tooth and implant sites (*p* < 0.001). The highest inflammatory values were observed in the sulcular epithelium around implants and in the junctional epithelium around teeth (Table S1). Fibroblast density was likewise significantly affected by the tissue region (*p* < 0.001), with the junctional epithelium exhibiting the highest values at both sites.

**FIGURE 6:**
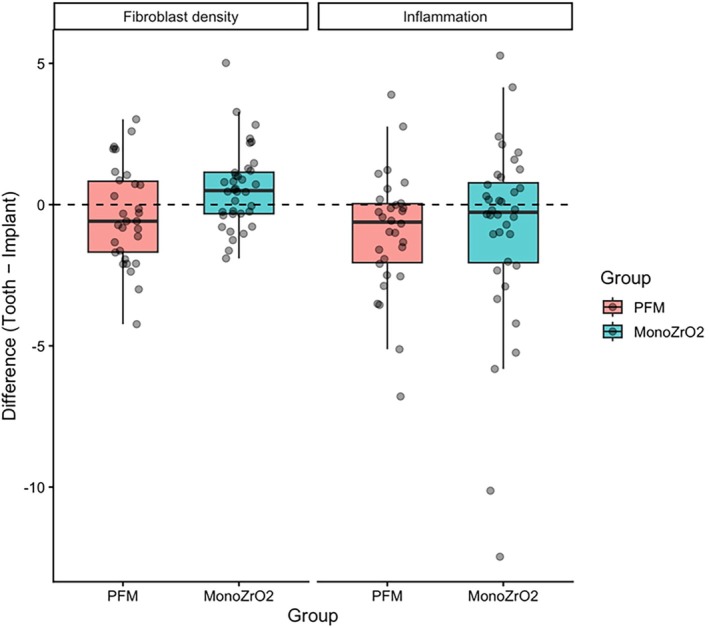
Within‐patient differences (tooth − implant) for fibroblast density and inflammation. Each point represents one patient. Positive values indicate higher values at tooth sites, while negative values indicate higher values at implant sites.

Within‐patient analyses (Table 3, Figure 6) supported the mixed‐effects model findings. Fibroblast density was significantly higher at the tooth than implant sites within the ZrO_2_ group (*p* = 0.027). In contrast, inflammatory infiltrate did not differ significantly between restorative materials (*p* = 0.373), although implant sites in the PFM group exhibited higher inflammatory values than tooth sites (*p* = 0.011). Sensitivity analyses adjusting for age, baseline keratinised tissue width and baseline mucositis yielded materially unchanged results, and none of these covariates showed significant associations with histological outcomes (Table S2).

**TABLE 3:** Within‐patient comparison of histological outcomes between tooth and implant sites according to restorative material.

Outcome	Material	Median difference (tooth − implant) (Q1–Q3)	Paired Wilcoxon *p*	Between‐material *p*
Fibroblast density (%)	PFM	−0.59 (−1.68 to 0.83)	0.262	0.019*
	ZrO_2_	0.49 (−0.32 to 1.15)	0.027*	
Inflammation (%)	PFM	−0.62 (−2.05 to 0.03)	0.011*	0.373
	ZrO_2_	−0.27 (−2.05 to 0.77)	0.252	

* *p* < 0.05.

## 4 Discussion

As a pre‐defined biological secondary outcome of the parent RCT, the present 6‐month clinical and histological assessment was intended to characterise early peri‐implant tissue adaptation following the insertion of monolithic zirconia crowns bonded to titanium bases or PFM restorations, and to compare these findings with the adjacent natural tooth. The main findings were that clinical outcomes remained stable from baseline to 6 months in both groups, with no clear restorative material–related differences. BOP differed between tooth and implant sites, with implant sites showing slightly higher values than tooth sites. Histological outcomes mainly differed by the tissue location and by site‐related interactions, whereas the restorative material alone did not significantly affect the extent of the inflammatory infiltrate or the fibroblast density.

Clinical studies reporting on histological findings of the peri‐implant soft tissues adjacent to zirconia‐based prosthetic components remain limited. Available human (Sampatanukul et al. 2018; Thoma, Sailer, et al. 2018; Thoma, Wolleb, et al. 2018; van Brakel et al. 2012; Vigolo et al. 2006) and preclinical evidence (Abrahamsson et al. 1998; Welander et al. 2008) generally suggest comparable soft‐tissue responses among titanium, zirconia and gold‐based components under controlled conditions, with only isolated preclinical findings indicating a less favourable mucosal attachment to porcelain‐fused‐to‐gold surfaces (Abrahamsson et al. 1998). In the present study, in addition to recording clinical parameters at both implant and adjacent tooth sites, paired soft‐tissue biopsies were obtained from the peri‐implant and the adjacent periodontal tissues within the same patient. This design provided paired clinical and histological data on the early soft‐tissue response to two restorative materials while largely minimising the confounding influence of inter‐individual differences in oral hygiene and host inflammatory susceptibility.

Clinically, both groups maintained stable peri‐implant soft‐tissue conditions over the 6‐month observation period. Keratinised tissue width, probing depth, plaque control record, BOP and marginal bone level changes showed only minor changes over time, and overall outcomes were comparable between the two groups. These findings are consistent with previous clinical studies indicating that both zirconia‐ and metal‐based implant‐supported reconstructions can maintain favourable peri‐implant tissue conditions, at least in the short term (Shen et al. 2022; Zhang et al. 2023). The mixed‐effects model showed that BOP was significantly higher at the implant than at adjacent tooth sites. This may reflect a greater tendency towards bleeding around implants or, at least in part, a false‐positive response to probing, as peri‐implant probing has been reported to be more sensitive than probing around natural teeth (Gerber et al. 2009). In addition, the lower cellularity and vascularity of peri‐implant connective tissue, together with the parallel arrangement of collagen fibres, may render peri‐implant soft tissues more susceptible to inflammation and its progression (Ivanovski and Lee 2018). In contrast, neither restorative material nor time significantly affected BOP, suggesting that early soft‐tissue bleeding is more closely related to the biological characteristics of peri‐implant tissues than to the restorative material itself.

Histological outcomes were mainly determined by the tissue region. In the present study, the inflammatory infiltrate showed a site‐dependent distribution pattern, being most pronounced in the sulcular epithelium (SE) around implants and in the junctional epithelium (JE) around natural teeth, while the fibroblast density was highest in the JE at both sites. This distribution may be related to the microbiota present in the peri‐implant biofilm (Penarrocha‐Oltra et al. 2016). Furthermore, the peri‐implant sulcus is generally wider, extending from approximately 1 mm around natural teeth to 3–4 mm around implants, which may alter the local host–biofilm interactions (Atsuta et al. 2016). However, the present findings differ from those of a previous histological study (Thoma, Wolleb, et al. 2018), in which significantly fewer inflammatory cells and fibroblasts/fibrocytes were observed in SE than JE and connective tissue, with inflammatory cells being mainly located within and adjacent to JE. One possible explanation for the discrepancy is the difference in biopsy harvesting protocols. In the present study, biopsies of both the peri‐implant mucosa and the adjacent gingiva of the natural tooth were harvested interproximally, whereas in the previous study biopsy samples were obtained from the palatal or lingual aspect of the implants. This difference in the tissue sampling location may have influenced the relative representation of epithelial and connective tissue compartments in the histological sections.

A smoother restorative surface has been suggested to promote more favourable soft‐tissue sealing and reduce bacterial colonisation (Sivaraman et al. 2018). Nevertheless, in the present study, the restorative material alone did not significantly influence either the inflammatory infiltrate or the fibroblast density. Although within‐patient comparisons revealed that the fibroblast density was significantly higher at the tooth than at implant sites in the monolithic zirconia group, no significant tooth–implant difference was detected in the PFM group. Similarly, for the inflammatory infiltrate, implant sites showed significantly higher values than tooth sites within the PFM group; however, this pattern did not differ significantly from that observed in the monolithic zirconia group. One possible explanation is that the histological evaluation was performed only once, that is, at 6 months after crown insertion. Peri‐implant soft‐tissue healing is a dynamic process, with early inflammatory changes potentially occurring within 4–12 weeks (Choe et al. 2024; Guarnieri et al. 2022; Salvi et al. 2015), whereas clinically stable and biologically meaningful outcomes may require longer term observation, often beyond 1 year.

Baseline width of keratinised tissue showed a numerical difference between groups at tooth sites. Sensitivity analyses including age, baseline keratinised tissue width and baseline peri‐implant mucositis status as covariates did not materially alter the histological findings (Table S2). The evidence on the long‐term benefit of keratinised mucosa for peri‐implant stability remains inconclusive; this variable was assessed as a descriptive secondary parameter.

Several limitations of the present study should be acknowledged. First, although the histological assessment was a pre‐specified biological secondary outcome of the parent RCT, the sample size calculation was based on the primary technical complication rate. Therefore, the findings should be interpreted as exploratory evidence suggesting no clear material‐related differences in early peri‐implant tissue response, rather than definitive evidence of equivalence. Second, H&E staining was the pre‐specified histological method (Abrahamsson et al. 1998). Although inflammatory cell subtype differentiation was not performed, this approach enabled standardised evaluation of the peri‐implant soft‐tissue architecture. Future studies incorporating immunohistochemical characterisation may provide additional biological insights.

## 5 Conclusion

Both monolithic zirconia and PFM crowns demonstrated stable short‐term peri‐implant clinical, radiographic and histological outcomes at 6 months, with no significant difference between restorative materials. Within the limitations of this exploratory analysis, the peri‐implant tissue response appeared to be influenced more strongly by the anatomical site and tissue region than by the restorative material itself.

## Author Contributions

**Yifan Zhang:** data curation, writing – original draft, funding. **Sven Mühlemann:** conceptualisation, investigation, resources, funding. **Aspasia Pachiou:** data curation, methodology, formal analysis. **Stephanie Astrid Stalder:** investigation, technical support. **Ronald E. Jung:** supervision, resources, funding. **Ye Lin:** resources, supervision, validation. **Daniel S. Thoma:** conceptualisation, supervision, resources; writing – review and editing.

## Funding

This study was supported by a grant (No. 937_2014) from the ITI Foundation and by the Clinic of Reconstructive Dentistry, Center of Dental Medicine, University of Zurich, Switzerland. Dr. Zhang was additionally supported by the National Natural Science Foundation of China (No. 82401062). Study materials, including implants and abutments, were provided by Institut Straumann AG.

## Ethics Statement

This study was officially approved by the local ethical committee (PB_2016‐01977); all persons involved had provided their informed consent prior to inclusion in the study.

## Conflicts of Interest

Institut Straumann AG provided study materials (implants and abutments) as in‐kind support; this constitutes a declarable interest. The funding body had no role in the study design, data collection, analysis, interpretation or manuscript preparation. The ITI Foundation provided unrestricted financial support. The other authors declare no conflicts of interest.

## Supporting information


**Table S1:** Estimated marginal means and post hoc comparisons from mixed‐effects models.
**Table S2:** Sensitivity analysis.

## Data Availability

The data supporting this study's findings are available from the corresponding author upon reasonable request.
